# Endometrial compaction can improve assisted reproductive technology outcomes in frozen-thawed embryo transfer cycles using hormone replacement therapy: A cross-sectional study

**DOI:** 10.18502/ijrm.v23i2.18484

**Published:** 2025-05-01

**Authors:** Shahrzad Moeinaddini, Saeideh Dashti, Zahra Amini Majomerd, Nooshin Hatamizadeh

**Affiliations:** Research and Clinical Center for Infertility, Yazd Reproductive Sciences Institute, Shahid Sadoughi University of Medical Sciences, Yazd, Iran.

**Keywords:** Assisted reproductive technology, Compaction, Endometrium, Embryo transfer, ART outcome.

## Abstract

**Background:**

Endometrial compaction (EC) is an ultrasound evaluation method that may predict assisted reproductive technology outcomes.

**Objective:**

This study aimed to assess the impact of EC on assisted reproductive technology outcomes in frozen embryo transfer cycles with hormone replacement therapy.

**Materials and Methods:**

In this cross-sectional study, 100 women who underwent first or second frozen embryo transfer cycle at Yazd Reproductive Sciences Institute, Yazd, Iran from June to October 2024 were included. Endometrial thickness was compared between the day of starting progesterone and embryo transfer day. Then participants were divided into 2 groups, no compaction and compaction group. Biochemical, clinical, and ongoing pregnancy rates (OPR) were assessed between the 2 groups.

**Results:**

Statistically significant differences were observed in biochemical, clinical, and OPR between the compaction and no compaction groups. Logistic regression analysis demonstrated significantly higher pregnancy rates in EC 10–15% and > 15%. We found a significant influence of EC 10–15% (p = 0.02, p = 0.01, p = 0.01), and EC > 15% (p = 0.002, p = 0.001, and p = 0.002) on biochemical, clinical, and OPR, respectively.

**Conclusion:**

EC after progesterone administration in hormone replacement therapy-frozen embryo transfer cycles can increase biochemical, clinical, and OPR. The percentage of EC changes also influence the outcomes of these cycles.

## 1. Introduction

The onset of pregnancy is signified by the embryo's implantation into the uterine, which is a pivotal event in the progression of pregnancy (1, 2). Despite the consistent improvement in success rates of assisted reproductive technologies (ARTs) since the first in vitro fertilization (IVF) procedure in 1978, implantation failure (IF) is observed in approximately 32–51% of all embryo transfers (3–7). IF may be attributable to several factors, including immunological processes, thrombophilias, endometrial receptivity, anatomical abnormalities, male factors, and embryo aneuploidy. Understanding the etiology of IF is essential for developing personalized treatment strategies (8, 9).

Endometrial receptivity has been demonstrated as a determining factor in the success of IVF procedures, which can be influenced by endometrial thickness (10, 11). The use of ultrasound technology for endometrial assessment has been indicated as a potential alternative approach for predicting endometrial receptivity and pregnancy outcomes (12). Some studies showed that endometrial thickness (ET) < 8 mm at the time of triggering in fresh IVF-embryo transfer cycles or < 7 mm at the end of the estrogenic-only phase in hormonally prepared frozen-thaw embryo transfer (FET) cycles was related to significantly lower live birth rates (13). An ultrasound evaluation of the endometrium, designated as endometrial compaction (EC), has been introduced. This term was defined as a decline in the ET between the end of the estrogen-only phase and the day of embryo transfer (14). In natural ovulatory cycles, the endometrial thinning is typically observed to be 1–1.5 mm, representing an average of 10–15% approximately 5 days after ovulation. Furthermore, EC following progesterone administration in artificial cycle FETs was associated with a higher rate of ongoing pregnancy than cycles without compaction, which may be an effective factor for evaluating endometrial receptivity (15).

Many studies have investigated that it is the relationship between EC and pregnancy outcomes (2, 3, 16, 17). According to conflicting results and the importance of EC following progesterone administration in ART outcomes in FET cycles, this study aimed to assess the impact of EC on ART outcomes in hormone replacement therapy (HRT)-FET cycles. We also calculated the percentage of EC and investigated its association with ART outcomes in these cycles.

## 2. Materials and Methods

### Study design and participants

This cross-sectional study was performed involving women aged between 20 and 40 yr who were candidates for first or second HRT-FET cycles between June and October 2024 at the Yazd Reproductive Sciences Institute, Yazd, Iran. Participants were excluded if they had a body mass index > 35, repeated IF ≥ 3, recurrent pregnancy loss ≥ 3, endometriosis, uterine abnormalities, severe male factor infertility, and lack of high-quality embryos (classified as grade C or D) on transfer day. Additionally, women with an ET < 7 mm on the 12 ${ߐ}^{\text{th}}$ or 13 ${ߐ}^{\text{th}}$ day of the menstrual cycle were also excluded from the study.

### Treatment protocol

Women were admitted for transvaginal ultrasound (TVUS) examination on the second day of their menstrual cycle by an infertility fellowship. On the same day, all participants commenced oral administration of 2 mg estradiol valerate (Aburaihan Pharmaceutical Co., Tehran, Iran) 3 times daily. ET and endometrial pattern were assessed via TVUS on the 12 ${ߐ}^{\text{th}}$ or 13 ${ߐ}^{\text{th}}$ day of the menstrual cycle. If the ET was ≥ 7 mm, luteal phase support was initiated with oral Dydrogesterone (Atipharmed Pharmaceutical Co., Karaj, Iran) at a dosage of 10 mg twice daily, in combination with vaginal progesterone suppositories (Atipharmed Pharmaceutical Co., Karaj, Iran) at a dosage of 400 mg twice daily. This hormonal support was continued for 2 wk following embryo transfer. 3 days after progesterone initiation, TVUS was done to evaluate ET before embryo transfer. Only high-quality embryos (Graded A or B) were selected for transfer. Serum beta-human chorionic gonadotropin (β-hCG) levels were measured 2 wk post-transfer to assess pregnancy status, and a subsequent ultrasound examination was performed 2 wk later to confirm clinical pregnancy. In cases of confirmed pregnancy, luteal phase support was maintained till 12 ${ߐ}^{\text{th}}$ wk of gestation. All pregnancies were followed until the 20 ${ߐ}^{\text{th}}$ wk of gestation.

### ET assessment and definition of EC

On the day of progesterone administration, ET and endometrial pattern (trilaminar vs. iso/hyper-echoic) were recorded. ET was re-evaluated transvaginally on the morning of the embryo transfer day. All ultrasound assessments were performed by a single infertility fellowship and the same ultrasound equipment (Philips Affinity 70 Ultrasound Machine). Additionally, the infertility fellowship was blinded to the ET in progesterone administration day. Endometrial thickness was measured in mid-sagittal plane (1). Participants were divided into 2 groups according to the difference in ET between the day of progesterone administration and embryo transfer:

• The compaction group (the group in which the thickness of the endometrium on the day of embryo transfer was thinner than that of progesterone administration) (n = 54).
• The no compaction group (the group in which the ET on the day of embryo transfer remained either unchanged or increased compared to that of progesterone administration) (n = 46).

The percentage of EC was considered as the difference between the ET on the day of progesterone administration and the ET on the day of embryo transfer, divided by the ET on the day of progesterone administration. The compaction percentage was defined based on previous studies (2, 3), compaction group was delineated as: > 15%, 10–15%, 5–10%, and < 5%.

### Definition of outcomes

Clinical pregnancy was defined as the presence of intrauterine gestational sac with heart beat and ongoing pregnancy was defined as fetal heart activity at ≥ 12 wk of gestation (18). Biochemical pregnancy was defined as serum β-hCG levels ≥ 10 mIU/ml 2 wk after embryo transfer (19). Abortion was considered as pregnancy loss before 20 wk of gestation.

### Ethical Considerations

This study was approved by the Ethics Committee of Yazd Reproductive Sciences Institute, Shahid Sadoughi University of Medical Sciences, Yazd, Iran (Code: IR.SSU.RSI.REC.1403.004). Written informed consent was obtained from participants.

### Statistical Analysis

Categorical variables were presented as frequencies and percentages. Continuous variables distributed normally were expressed as the mean ± standard deviation. In contrast, continuous variables that did not conform to a normal distribution were expressed as the median and interquartile range. The normality of the variables was evaluated by utilizing the Shapiro-Wilk tests. The Chi-square or Fisher's exact test was used to compare categorical variables. The difference between quantitative and qualitative variables, with more than 2 groups, was tested by one-way ANOVA for parametric variables and Kruskal-Wallis for non-parametric variables. To study the effect of EC on ART outcomes, logistic regression analysis was used to calculate the odds ratios (ORs) of 95% confidence intervals (CIs). A significance level of 0.05 was accepted to evaluate the statistical significance. All statistical analysis was conducted on the IBM Statistical Package for the Social Sciences (IBM SPSS v.25; IBM Corp).

## 3. Results

**Figure 1:**
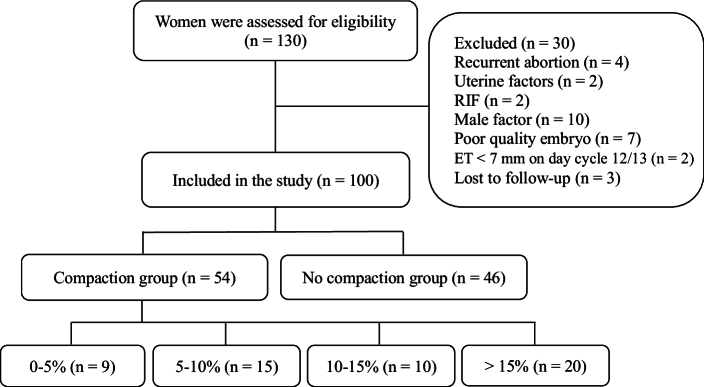
Flowchart showing enrollment of participants, ET: Endometrial thickness, RIF: Recurrent implantation failure.

Initially, 130 women were eligible to enter the study. Of them, 30 women were excluded due to exclusion criteria. Finally, 100 women aged between 20 and 40 yr were analyzed and divided into 2 groups: compaction group (n = 54) and no compaction group (n = 46) (Figure 1).

The participants were divided into 2 groups “compaction" or “no compaction" according to the difference in ET between the day of embryo transfer and the day of progesterone administration. Participants in the compaction group were subdivided into 4 subgroups based on the percentage of EC, which was defined as follows: < 5%, 5–10%, 10–15%, and > 15%. In all FET cycles, 46 women (46%) had no compaction, while 9 women (9%) had < 5%. Additionally, 15 women (15%) demonstrated EC 5–10%, 10 women (10%) EC 10–15%, and 20 women (20%) had EC > 15% on the day of embryo transfer.

Of baseline characteristics, there was no differences in the age of participants, retrieved oocyte, body mass index, duration of infertility, types and causes of infertility, anti-Mullerian hormone, gravidity, parity, number of prior abortions and transferred embryos (Table I).

The cycle characteristics (like endometrial patterns, type of ART) were comparable between the 2 groups. Statistically significant differences were observed in biochemical, clinical, and ongoing pregnancy rates (OPR) between the compaction and no compaction groups (Table II).

Subsequently, logistic regression was done to determine the effect of percentages of EC changes in the compaction group on ART outcome. The results demonstrated significantly higher biochemical, clinical, and OPR in EC 10–15% and above 15% compared to no compaction group. Hence, we found a significant influence of EC 10–15% (OR = 5.57; CI: 1.27–24.42; p = 0.02), (OR = 6.66; CI: 1.47–30.10; p = 0.01), (OR = 6.66; CI: 1.47–30.10; p = 0.01), and EC above 15% (OR = 6.81; CI: 2.06–22.45; p = 0.002), (OR = 8.14; CI: 2.38–27.87; p = 0.001), (OR = 6.66; CI: 1.95–22.73; p = 0.002) on biochemical, clinical and OPR, respectively (Table III).

**Table 1:** Baseline characteristics of variables with and without EC

**Variables**		**Compaction (%)**				**No compaction (n = 46)**	**P-value**
		**0–5** **(n = 9)**	**5–10** **(n = 15)**	**10–15** **(n = 10)**	**> 15** **(n = 20)**		
Age at transfer (yr)*		30.56 ± 6.02 (33, 11)	32.73 ± 7.35 (36, 12)	32.50 ± 4.50 (33.50, 8)	32.90 ± 5.91 (33.50, 10)	32.07 ± 5.15 (32, 9)	0.43
Age at oocyte retrieval (yr)*		29.33 ± 4.92 (31, 9)	30.53 ± 7.60 (32, 12)	31.10 ± 4.28 (31.50, 7)	31.40 ± 5.80 (31.50, 6)	30.13 ± 5.74 (30, 9)	0.63
Body mass index (kg/m^2^)*		26.62 ± 4.03 (27.40, 5.40)	27.18 ± 4.20 (26.20, 6)	26.88 ± 3.01 (25.75, 5.20)	25.15 ± 4.57 (22.85, 8.10)	27.42 ± 4.01 (27.45, 5.50)	0.58
Infertility duration (yr)*		5.33 ± 2.91 (4, 5)	7.10 ± 4.48 (7, 7)	7.70 ± 5.12 (7, 9)	8.30 ± 4.07 (7.50, 6)	6.59 ± 3.77 (6, 5)	0.58
Types of infertility**							
	**Primary**	6 (66.7)	11 (73.3)	7 (70)	16 (80)	29 (63)	0.72
	**Secondary**	3 (33.3)	4 (26.7)	3 (30)	4 (20)	17 (37)	
Causes of infertility**							
	**Male factor**	2 (22.2)	5 (33.3)	2 (20)	11 (55)	15 (32.6)	0.71
	**Ovulation dysfunction**	4 (44.4)	3 (20)	4 (40)	4 (20)	10 (21.7)	
	**Low ovarian reserve**	0 (0)	0 (0)	0 (0)	0 (0)	1 (2.2)	
	**Unknown**	0 (0)	2 (13.3)	1 (10)	0 (0)	2 (4.3)	
	**Mixed**	3 (33.3)	5 (33.3)	3 (30)	5 (25)	18 (39.1)	
AMH (ng/ml)*		8.07 ± 4.97 (7.90, 6.50)	4.78 ± 3.87 (3.00, 5.90)	4.28 ± 2.60 (3.75, 3.90)	5.75 ± 3.62 (5.15, 6.70)	5.37 ± 4.11 (3.90, 3.90)	0.08
Gravidity*		0.44 ± 0.52 (0.00, 1.00)	0.47 ± 0.64 (0.00, 1.00)	0.20 ± 0.42 (0.00, 0.00)	0.25 ± 0.55 (0.00, 0.00)	0.50 ± 0.78 (0.00, 1.00)	0.83
Parity*		0.33 ± 0.50 (0.00, 1.00)	0.27 ± 0.45 (0.00, 1.00)	0.20 ± 0.42 (0.00, 0.00)	0.20 ± 0.41 (0.00, 0.00)	0.35 ± 0.56 (0.00, 1.00)	0.94
Live births*		0.33 ± 0.50 (0.00, 1.00)	0.20 ± 0.41 (0.00, 0.00)	0.20 ± 0.42 (0.00, 0.00)	0.20 ± 0.41 (0.00, 0.00)	0.35 ± 0.56 (0.00, 1.00)	0.93
Previous abortions*		0.12 ± 0.36 (0.00, 0.00)	0.27 ± 0.59 (0.00, 0.00)	0.07 ± 0.20 (0.00, 1.00)	0.05 ± 0.22 (0.00, 0.00)	0.15 ± 0.42 (0.00, 0.00)	0.63
Implantation failures*		0.22 ± 0.44 (0.00, 1.00)	0.40 ± 0.50 (0.00, 1.00)	0.70 ± 0.82 (0.50, 1.00)	0.85 ± 0.74 (1.00, 1.00)	0.67 ± 0.70 (1.00, 1.00)	0.06
Transferred embryo*		1.89 ± 0.33 (2.00, 0.00)	1.80 ± 0.56 (2.00, 1.00)	1.80 ± 0.63 (2.00, 1.00)	1.90 ± 0.30 (2.00, 0.00)	1.89 ± 0.37 (2.00, 0.00)	0.90
*Data presented as Mean ± SD (median, interquartile range), One-way ANOVA, and Kruskal Wallis test for the comparison variables between groups. **Data presented as n (%), Chi-square test. EC: Endometrial compaction, AMH: Anti-Mullerian hormone							

**Table 2:** Cycle characteristics of variables with and without EC

**Variables**		**Compaction (%)**				**No compaction (n = 46)**	**P-value**
		**0–5 (n = 9)**	**5–10 (n = 15)**	**10–15 (n = 10)**	** > 15 (n = 20)**		
**Endometrial pattern**							
	**3-line**	7 (77.8)	12 (80)	7 (70)	14 (70)	36 (78.3)	0.66
	**Echogenic**	1 (11.1)	1 (6.7)	1 (10)	5 (25)	8 (17.4)	
	**Isoechoic**	1 (11.1)	2 (13.3)	2 (20)	1 (5)	2 (4.3)	
**Types of ART**							
	**IVF**	0 (0)	0 (0)	0 (0)	1 (5)	2 (4.3)	0.53
	**ICSI**	6 (66.7)	9 (60)	5 (50)	15 (75)	35 (76.1)	
	**IVF+ICSI**	3 (33.3)	6 (40)	5 (50)	4 (20)	9 (19.6)	
Abortion rate*		1/2 (50)	1/3 (33.3)	1/5 (20)	1/11 (9.1)	1/6 (16.7)	0.24
**Biochemical pregnancy rate**		2 (22.2)	3 (20)	5 (50)	11 (55)	7 (15.2)	0.007
**Clinical pregnancy rate**		2 (22.2)	3 (20)	5 (50)	11 (55)	6 (13)	0.004
**Ongoing pregnancy rate**		1 (11.1)	2 (13.3)	5 (50)	10 (50)	6 (13)	0.003
Data presented as n (%), Chi-square or Fisher's exact test for the comparison variables between groups. *Data presented as number of abortion per clinical pregnancy × 100. EC: Endometrial compaction, ART: Assisted reproductive technology, IVF: In vitro fertilization, ICSI: Intra-cytoplasmic sperm injection							

**Table 3:** Associations of pregnancy outcome with EC

**EC (%)**		**OR (95% CI)**	**P-value**
**Biochemical pregnancy**			
	**0–5**	1.59 (0.27–9.30)	0.60
	**5–10**	1.39 (0.31–6.23)	0.66
	**10–15**	5.57 (1.27–24.42)	0.02
	** > 15**	6.81 (2.06–22.45)	0.002
	**No compaction**	Reference	—
**Clinical pregnancy**			
	**0–5**	1.90 (0.31–11.41)	0.48
	**5–10**	1.66 (0.36–7.68)	0.51
	**10–15**	6.66 (1.47–30.10)	0.01
	** > 15**	8.14 (2.38–27.87)	0.001
	**No compaction**	Reference	—
**Ongoing pregnancy**			
	**0–5**	0.83 (0.08–7.89)	0.87
	**5–10**	1.02 (0.18–5.71)	0.97
	**10–15**	6.66 (1.47–30.10)	0.01
	** > 15**	6.66 (1.95–22.73)	0.002
	**No compaction**	Reference	—
The p-value and odds ratio were calculated using logistic regression. EC: Endometrial compaction, OR: Odd ratio, CI: Confidence interval			

## 4. Discussion

The ET at the end of the estrogen-only phase and on the day of embryo transfer may influence ART outcomes in HRT-FET cycles. Furthermore, the EC following the administration of progesterone may be a determining factor in the outcome of these cycles. However, the percentage of EC changes may also influence the outcome of HRT-FET cycles. Our study indicated that EC was associated with an increased rate of biochemical, clinical, and ongoing pregnancy, with a significant association observed in EC above 10% in HRT-FET cycles.

In a normal menstrual cycle, the proliferative phase is characterized by endometrial thickening, which occurs in response to increased estrogen levels. Following ovulation and the subsequent secretion of progesterone, the proliferation of the endometrium ceases. However, the proliferation of glands and vessels continues, developing a compact secretory endometrium (20). The failure of EC or increase in thickness following the secretion of progesterone may be attributed to an inadequate response of the endometrium to the progesterone. This disproportionate response can be related to some factors, including the deficiency or resistance of endometrial progesterone receptors, insufficient serum levels of progesterone, impaired proliferation of the follicular phase endometrium, and chronic inflammation. Therefore, the compaction of the endometrium during the secretory phase may indicate favorable conditions for embryo implantation (9). The role of endometrial factors and their effect on ART outcomes remains unclear. Several studies have demonstrated that the compaction of the endometrium and the decrease in ET following the secretion of progesterone in the secretory phase effectively improve the fertility outcome in the FET cycles.

Nevertheless, the results of the studies have not been consistent in terms of ART outcomes. EC may increase live birth rates in artificial FET cycles. Yaprak et al. demonstrated in their study that the chance of LBR was more than 3 times in artificial FET cycles with EC (21). Our study showed that the difference in ET on the day of progesterone administration and embryo transfer in the compaction group was significantly correlated with pregnancy outcomes.

An increase in the ET change ratio has been demonstrated to enhance the clinical pregnancy rate (CPR) in FET cycles. A cohort study investigated the impact of changes in ET following progesterone administration on the CPR in single frozen-thawed euploid blastocyst transfer cycles using natural cycles. They concluded that although 25% of women exhibited EC, this was associated with increased CPR. Furthermore, it was observed that when the percentage of EC reached 10%, a significant increase was observed in the CPR (22). Moreover, EC has been demonstrated to enhance the probability of OPR. A study investigating ET after progesterone administration demonstrated that endometrial changes following progesterone administration can improve pregnancy outcomes of thawed blastocyst transfer in artificial FET cycles. The study showed that EC was associated with an increased OPR, particularly in cases with compaction > 10% (15). In a cohort study, Ju et al. examined the effect of EC following progesterone administration on the outcome of artificial FET cycles. They reported that the CPR was increased significantly in cases with EC exceeding 5% on the day of embryo transfer (23). Another study additionally described the role of EC of > 5% in a modified natural cycle euploid embryo transfer in improving the outcome of the ongoing pregnancy (24). A systematic review investigating the role of EC on ART outcomes, demonstrated that EC may significantly enhance CPR and OPR. However, LBR represents the most meaningful outcome of pregnancy. Consequently, the use of EC to stratify women in clinical practice is currently unwarranted.

Nevertheless, further investigation is required to determine the potential effect of EC as a noninvasive predictor. Therefore, future clinical trials recommended to include LBR as the primary outcome (25). It is noteworthy that our study demonstrated that EC following the administration of progesterone has a beneficial impact on the outcome of HRT-FET cycles, with an increase in the biochemical pregnancy rates, CPR, and, OPR. These findings align with those of previous studies in this field.

While several studies have indicated that EC may improve ART outcomes, the precise percentage of EC that improves outcomes remains debatable. A cohort study conducted on 234 women who underwent frozen euploid embryo transfer demonstrated that EC can significantly impact on the pregnancy rate. The OPR increased significantly in women who had an EC of more than 5% after progesterone administration. The OPR demonstrated an increase of over 50% compared to the no compaction group. They demonstrated that EC at 5%, 10%, 15%, and 20% cut-offs, resulted in a significant increase in the OPR. At cut-off values above 10%, the results demonstrated acceptable specificity (67.1%) but relatively low sensitivity (47.6%). The highest sensitivity was observed at the 5% cut-off point (52.4%), while the highest specificity was noted at the 20% cut-off point (83.9%) (26). Haas et al. showed that EC more than 5% after progesterone administration in FET cycles improves pregnancy rates significantly compared to the no EC group. They indicated a significant correlation between EC at cut-off above 5%, 10%, and 15% with OPR. EC, with a cut-off of more than 10%, had a sensitivity of 45.5% and a specificity of 79.2%. The highest sensitivity reported was 59.1% at the 5% cut-off, and the highest specificity at the 15% cut-off was 87.4% (27). In another study, the role of EC in the natural FET cycle was examined, and the relationship between EC and OPR and CPR was investigated. The study demonstrated that EC of over 5–10% between the day of ovulation and the day of embryo transfer can increase both OPR and CPR. However, an EC of over 15% only increases the OPR. In cases of EC above 10%, the sensitivity for CPR and OPR was reported to be 48% and 44%, respectively, with specificities of 78% and 75%. It was found that the highest sensitivity for both the CPR and OPR was associated with EC above 5%, while the highest specificity was related to EC above 15% (28).

Our findings indicate that EC more than 10% was associated with increased biochemical pregnancy, CPR, and OPRs. The probability of a positive β-hCG test in cases of EC between 10 and 15% and > 15% increases by more than 5 and 6 times, respectively. Furthermore, the probability of observing the fetal heart in the TVUS at 5 wk after embryo transfer in 2 distinct cut-off ranges (10–15% and > 15%) is more than 6 and 8 times, respectively. Additionally, the probability of ongoing pregnancy in ultrasound at 12 wk of gestation increases by more than 6-fold in both cut-off ranges. Accordingly, our study indicates an increased probability of biochemical, clinical, and ongoing pregnancy can be expected in cases where EC is more than 10% compacted.

### Strengths and limitations

The present study was subject to some limitations. One of the limitations of the study was its single-center nature. Furthermore, the follow-up of the participants was conducted until the ongoing pregnancy, which precluded the LBR as a pregnancy outcome. Additionally, preimplantation genetic testing was not routine at our center. However, we attempted to mitigate this limitation by selecting high-quality embryos and minimizing the impact of embryo quality on the pregnancy rate. A low sample size of the study was also a potential limitation.

## 5. Conclusion

EC after progesterone administration in HRT-FET cycles may increase biochemical pregnancy rates, CPR, and OPR. EC above 10% may be associated with an increased rate of pregnancy. Nevertheless, further research through more extensive studies is recommended to determine the significance of the percentage of EC.

##  Data Availability

The data and materials of the current study are available from the corresponding author upon reasonable request.

##  Author Contributions

Sh. Moeinaddini: Acquisition of data, and interpretation of data, drafting of the manuscript, technical and material support. S. Dashti: Study concept, design, and supervision, drafting of the manuscript, technical and material support. Z. Amini Majomerd: Acquisition of data, drafting of the manuscript, technical and material support. N. Hatamizadeh: Acquisition of data, drafting of the manuscript, technical and material support. All authors read and approved the final version of the manuscript.

##  Conflict of Interest

The authors declare that there is no conflict of interest.
